# Supplementary material for: Targeting ADAR stability: a novel approach to combat glioblastoma
Source: Front Oncol. 2026 Aug 6;16:1827793. doi: 10.3389/fonc.2026.1827793 (PMC13489870; doi:10.3389/fonc.2026.1827793)
Supplement: Supplementary file 1 [file SupplementaryFile1.zip › Supplementary file 1/Pentose_phosphate_pathway.pdf]

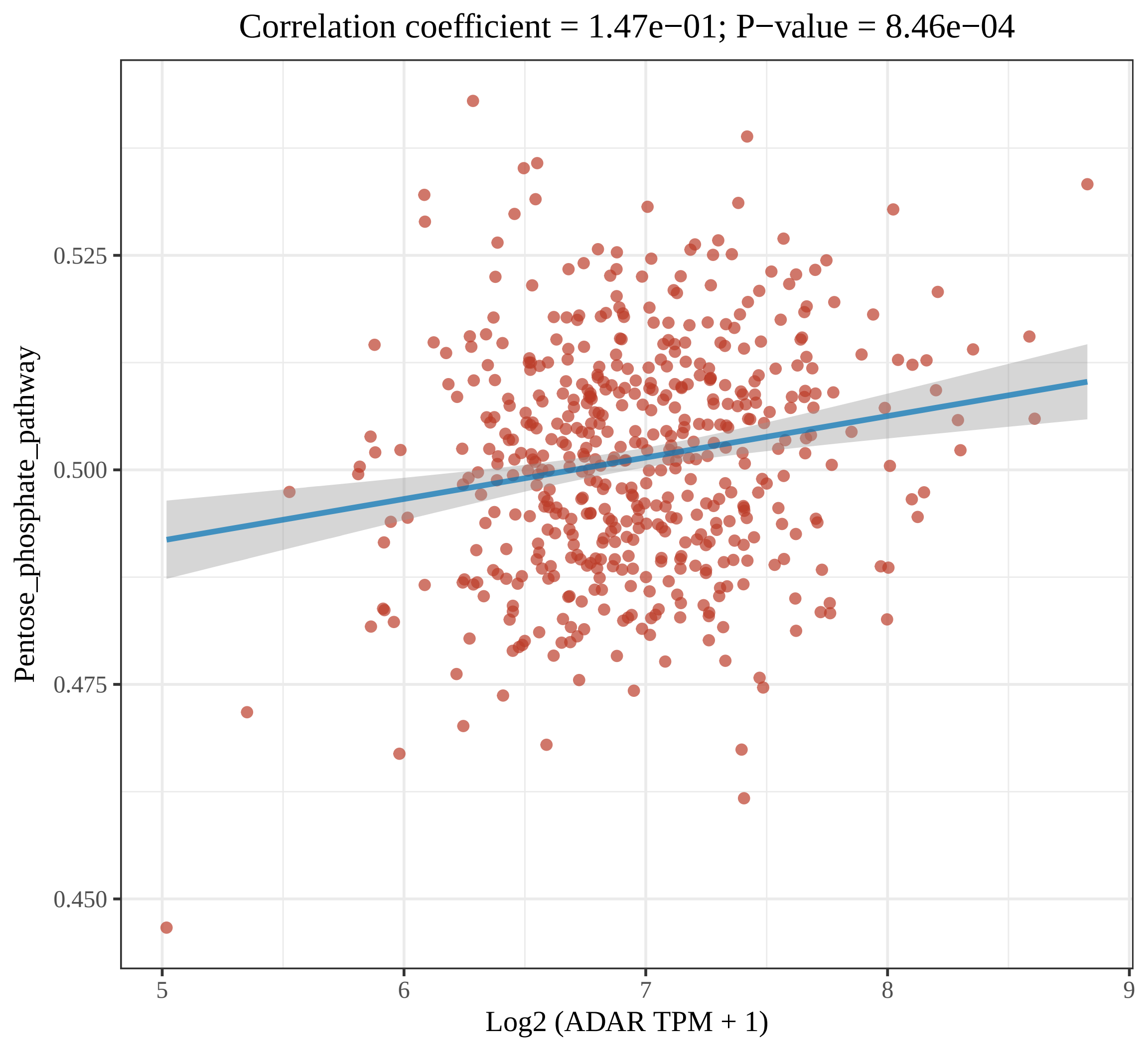

Correlation coefficient = 1.47e−01; P−value = 8.46e−04
Pentose_phosphate_pathway
0.525
0.500
0.475
0.450
5
6
7
8
9
Log2 (ADAR TPM + 1)
